# The rVSV-EBOV vaccine provides limited cross-protection against Sudan virus in guinea pigs

**DOI:** 10.1038/s41541-023-00685-z

**Published:** 2023-06-10

**Authors:** Wenguang Cao, Shihua He, Guodong Liu, Helene Schulz, Karla Emeterio, Michael Chan, Kevin Tierney, Kim Azaransky, Geoff Soule, Nikesh Tailor, Abdjeleel Salawudeen, Rick Nichols, Joan Fusco, David Safronetz, Logan Banadyga

**Affiliations:** 1 Special Pathogens Program, National Microbiology Laboratory, Public Health Agency of Canada, Winnipeg, MB R3E 3R2 Canada; 2 Department of Medical Microbiology and Infectious Diseases, University of Manitoba, Winnipeg, MB R3E 0J9 Canada; 3 Public Health Vaccines, Cambridge, MA 02142 USA

**Keywords:** Live attenuated vaccines, Ebola virus

## Abstract

Recombinant vesicular stomatitis viruses (rVSVs) engineered to express heterologous viral glycoproteins have proven to be remarkably effective vaccines. Indeed, rVSV-EBOV, which expresses the Ebola virus (EBOV) glycoprotein, recently received clinical approval in the United States and Europe for its ability to prevent EBOV disease. Analogous rVSV vaccines expressing glycoproteins of different human-pathogenic filoviruses have also demonstrated efficacy in pre-clinical evaluations, yet these vaccines have not progressed far beyond research laboratories. In the wake of the most recent outbreak of Sudan virus (SUDV) in Uganda, the need for proven countermeasures was made even more acute. Here we demonstrate that an rVSV-based vaccine expressing the SUDV glycoprotein (rVSV-SUDV) generates a potent humoral immune response that protects guinea pigs from SUDV disease and death. Although the cross-protection generated by rVSV vaccines for different filoviruses is thought to be limited, we wondered whether rVSV-EBOV might also provide protection against SUDV, which is closely related to EBOV. Surprisingly, nearly 60% of guinea pigs that were vaccinated with rVSV-EBOV and challenged with SUDV survived, suggesting that rVSV-EBOV offers limited protection against SUDV, at least in the guinea pig model. These results were confirmed by a back-challenge experiment in which animals that had been vaccinated with rVSV-EBOV and survived EBOV challenge were inoculated with SUDV and survived. Whether these data are applicable to efficacy in humans is unknown, and they should therefore be interpreted cautiously. Nevertheless, this study confirms the potency of the rVSV-SUDV vaccine and highlights the potential for rVSV-EBOV to elicit a cross-protective immune response.

## Introduction

Filoviruses pose a significant threat to global public health^1^. Of the dozens of filoviruses that have so far been described, six of them are known to cause disease that is among the most severe viral illnesses observed in humans. Marburg virus (MARV) and Ravn virus (RAVV) both belong to the *Marburgvirus* genus and have been causing sporadic outbreaks mostly in Central Africa at least since the discovery of MARV in 1967. Moreover, MARV recently emerged for the first time in West Africa causing a small outbreak in 2021 and another in 2022^2,3^. Ebola virus (EBOV), Bundibugyo virus (BDBV), Taï Forest virus (TAFV), and Sudan virus (SUDV) all belong to the *Ebolavirus* genus and have collectively caused numerous severe outbreaks throughout Africa since the first recorded appearance of EBOV and SUDV in 1976. Indeed, EBOV was responsible for the 2013–2016 West African epidemic that sickened more than 28,000 people and killed nearly half of them^4^. The West African EBOV epidemic was followed by several others, including a large outbreak in the Democratic Republic of the Congo in 2018–2020 that killed over 2000 people^5^. After EBOV and MARV, SUDV has been responsible for the most outbreaks, with five of the previous six occurring in Uganda, including the largest SUDV outbreak on record, which caused 425 cases and 224 deaths^6^. On 11 January 2023, the Ugandan Ministry of Health declared the end of the most recent SUDV outbreak, which resulted in 142 confirmed cases and 55 deaths since the outbreak started on 20 September 2022^7^.

The 2013–2016 West African EBOV epidemic stimulated remarkable progress in the pre-clinical and clinical development of filovirus countermeasures, although much of the groundwork for this rapid advancement had already been laid in the preceding decades of basic research^8^. In 2004, a recombinantly engineered vesicular stomatitis virus (rVSV) was first used as a vaccine to protect against disease caused by EBOV^9^. This vaccine was generated by removing the endogenous VSV glycoprotein (G) gene and replacing it with the gene for the EBOV glycoprotein (GP), which is responsible for virion entry and fusion. The resulting live, attenuated chimeric virus—known as rVSV∆G-ZEBOV-GP or, simply, rVSV-EBOV—expresses EBOV GP as the sole viral protein on the surface of the VSV virion. rVSV-EBOV has proven to be a remarkably safe, immunogenic, and effective vaccine, with years of research culminating in its recent licensure (Ervebo, Merck) in the United States and Europe for the prevention of EBOV disease^10^. The rVSV vaccine platform has also been used to develop a number of other experimental vaccines analogous to rVSV-EBOV^11^, including a SUDV vaccine (rVSV-SUDV) and a Lassa virus vaccine (rVSV-LASV).

Despite the recent advances made in countermeasure development, and despite a research pipeline full of promising experimental vaccines and therapeutics, there are still no approved treatments or prophylactics available for any filovirus other than EBOV. To date, only three SUDV-specific vaccine candidates have progressed to the point of Phase I clinical trials^12^. Two chimpanzee adenovirus-vectored vaccines—cAd3 expressing SUDV GP^13^ and chAdOx1 expressing both EBOV and SUDV GP^14,15^—are among the most promising vaccine candidates, although the Ad26.ZEBOV/MVA-BN-Filo prime-boost vaccine may also prove useful. Indeed, Ad26.ZEBOV/MVA-BN-Filo, which is already approved for use against EBOV (Zabdeno and Mvabea, Johnson & Johnson), may also confer protection against SUDV, thanks to the inclusion of SUDV GP in the MVA-BN-Filo boost^16^. Nevertheless, Ad26.ZEBOV/MVA-BN-Filo requires two injections, with Ad26.ZEBOV administered initially and MVA-BN-Filo administered as a boost. As a result, this vaccine may not be the best choice for outbreak response. rVSV-based vaccines, on the other hand, have demonstrated exceptional efficacy, with protection elicited relatively rapidly after only a single dose. Thus, in an effort to support the pre-clinical development of an rVSV-based SUDV vaccine, we used our guinea pig model of SUDV infection to evaluate our experimental rVSV-SUDV (also referred to as rVSV∆G-SUDV-GP), which expresses the GP of SUDV variant Boneface in place of VSV G. Not surprisingly, our results confirm the effectiveness of this vaccine, which completely protected animals from SUDV disease and death, even when administered as a single injection at a relatively low dose. Not only do these data underscore the utility of rVSV-vectored vaccines against filoviruses, but they also lay a foundation for the future clinical development of this particular rVSV vaccine.

At the same time, however, we were also interested in knowing whether rVSV-EBOV was capable of providing cross-protection against SUDV challenge, since a readily available and clinically approved vaccine could have a meaningful benefit during an outbreak, even if it is only partially effective. Filoviruses are known to be serologically cross-reactive, and cross-reactivity may be higher among EBOV, SUDV, BDBV, and TAFV, which all belong to the *Ebolavirus* genus^17–19^. Indeed, the structural similarity between ebolavirus GPs is what has enabled the generation of broadly cross-reactive monoclonal antibodies, many of which were isolated from EBOV survivors but are capable of neutralizing multiple ebolaviruses^20^. However, while cross-protection may be possible in principle, limited reports with small numbers of nonhuman primates (NHPs) have suggested that rVSV-EBOV cannot protect against SUDV challenge^21,22^. We, therefore, sought to re-evaluate the cross-protective efficacy of rVSV-EBOV in guinea pigs challenged with guinea pig-adapted (GPA) SUDV. Surprisingly, we observed that nearly 60% (8/14) of the guinea pigs that had been vaccinated with rVSV-EBOV survived the challenge with SUDV, although most animals were not protected from disease. These results were confirmed by a back-challenge experiment in which rVSV-EBOV-immunized guinea pigs that had survived infection with GPA-EBOV were re-challenged with GPA-SUDV. Together, these data demonstrate that rVSV-EBOV is capable of eliciting an immune response that is cross-protective against SUDV in guinea pigs, but whether this phenomenon can be replicated in NHPs or humans remains to be determined.

## Results

### Immunization with rVSV-SUDV protects guinea pigs from lethal SUDV infection

**Fig. 1:**
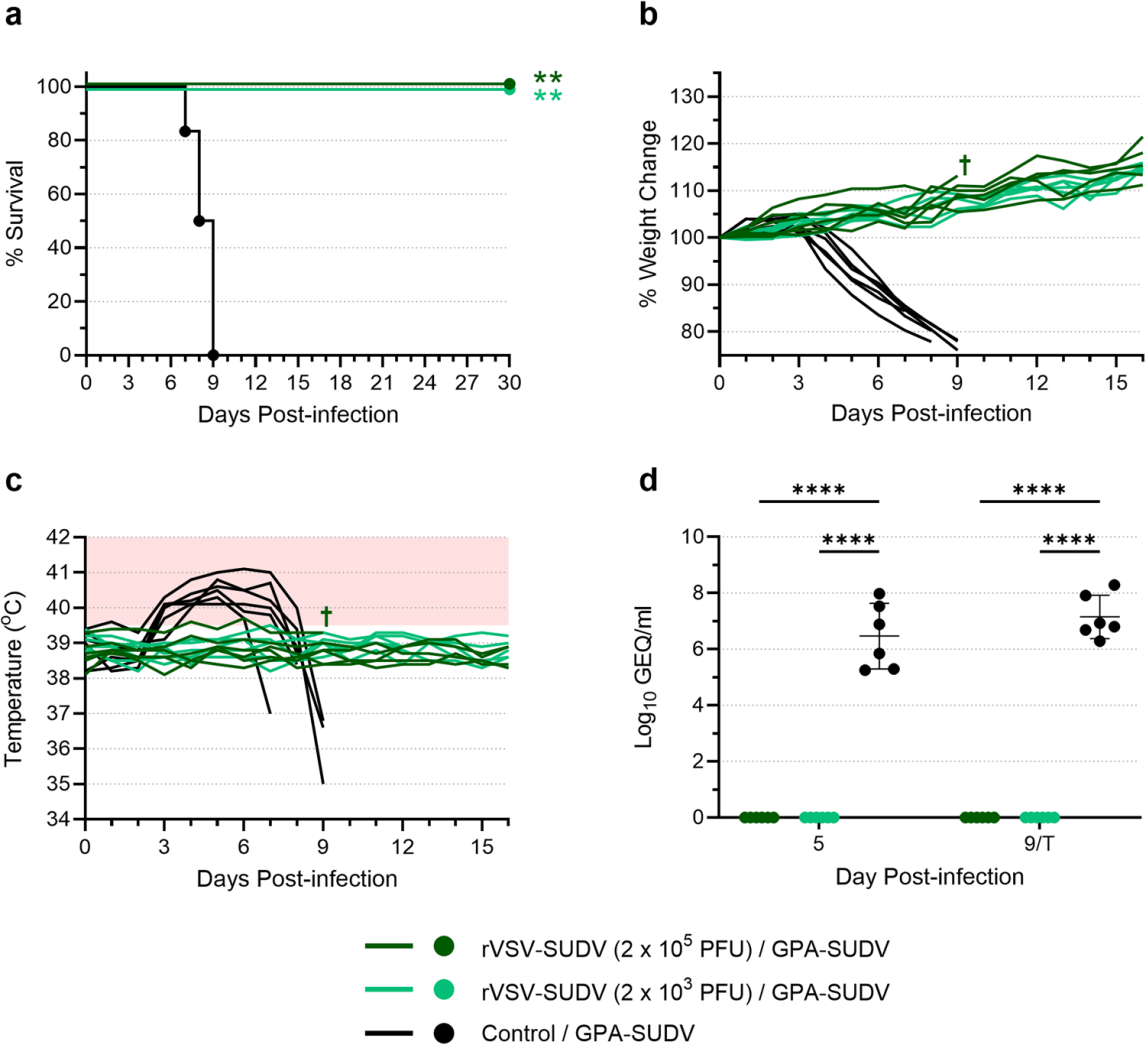
rVSV-SUDV protects against SUDV challenge in guinea pigs. Groups of six guinea pigs were vaccinated with rVSV-SUDV at a dose of either 2 × 10^5^ PFU or 2 × 10^3^ PFU. Control animals (*n* = 6) received an equivalent volume of saline. Twenty-eight days after vaccination, animals were challenged with 1000 LD_50_ of GPA-SUDV. Animals were monitored for survival (**a**), weight change (**b**), and body temperature (**c**). The area shaded pink in (**c**) highlights temperatures above 39.5 °C. Blood samples were obtained from each animal on day 5 post-infection and either day 9 post-infection or at the terminal time point (T) if it occurred before day 9. Samples were assessed for levels of virus RNA via RT-qPCR, and data are presented as Log_10_ genome equivalents (GEQ) per milliliter for each animal, with means and standard deviations indicated (**d**). A single animal (†) that was vaccinated with 2 × 10^5^ PFU rVSV-SUDV died during sampling on day 9; this animal did not exhibit signs of disease and is considered a survivor. Survival curves (**a**) were compared using the Log-Rank test with the Bonferroni correction for multiple comparisons; virus RNA levels (**d**) were compared using a two-way ANOVA with Tukey’s multiple comparison test. **p ≤ 0.01; ****p ≤ 0.0001.

To demonstrate the protective efficacy of rVSV-SUDV in the guinea pig model, two groups of six animals were immunized with either 2 × 10^5^ or 2 × 10^3^ PFU of rVSV-SUDV, and one group of 6 control animals was administered saline instead of vaccine. All animals were challenged with a uniformly lethal dose of GPA-SUDV on day 28 post-vaccination (Supplementary Fig. 1a). All 6 control animals developed severe disease and succumbed to SUDV infection within 7–9 days, following significant weight loss and fever, defined as a body temperature >39.5 °C for at least 2 consecutive days (Fig. 1a–c). Conversely, none of the vaccinated animals—regardless of vaccine dose—developed disease or succumbed to infection, with all animals consistently gaining weight and maintaining a normal body temperature throughout the study (Fig. 1a–c). A single vaccinated animal was lost during sampling on day 9 post-infection, but this was determined to be the result of a sampling accident. The animal itself was otherwise healthy and did not exhibit any signs of virus infection.

Levels of virus RNA in the blood were assessed by RT-qPCR for all animals on day 5 post-infection and again on either day 9 or the terminal time point (prior to euthanasia), if it occurred before day 9 (Fig. 1d). Remarkably, none of the immunized animals exhibited detectable levels of SUDV RNA, suggesting that viremia was prevented by immunization with rVSV-SUDV. In contrast, the control animals exhibited significantly higher levels of virus RNA on both day 5 and the terminal time point, reflective of the severe disease they experienced.

**Fig. 2:**
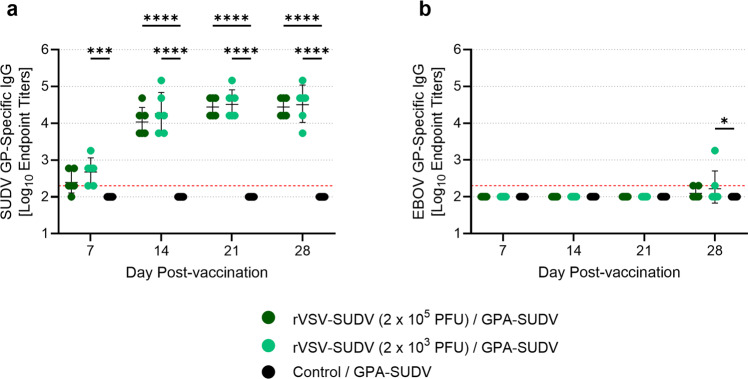
rVSV-SUDV elicits a robust humoral immune response. Serum samples were obtained from all animals on days 7, 14, 21, and 28 after vaccination with rVSV-SUDV. Samples were assessed for levels of SUDV GP-specific IgG (**a**) or EBOV GP-specific IgG (**b**) via ELISA. Data are presented as Log_10_ endpoint titers for each animal, with the geometric means and standard deviations indicated. The lower limit of detection is indicated with a red dashed line. Mean IgG (**a**, **b**) levels were compared using a two-way ANOVA with Tukey’s multiple comparison test. *p ≤ 0.05; ***p ≤ 0.001; ****p ≤ 0.0001.

Animals were likely protected from SUDV disease by the potent humoral immune response elicited by vaccination. Indeed, an ELISA revealed low levels of SUDV GP-specific IgG as early as 7 days post-vaccination, with endpoint titers in all animals increasing by day 14 and remaining high until the time of SUDV challenge (Fig. 2a). By day 28 post-vaccination, the geometric mean endpoint titers for all vaccinated animals were around 4.5 Log_10_. As expected, the control animals did not mount an IgG response to SUDV GP. Interestingly, immunization with rVSV-SUDV did not elicit appreciable levels of EBOV GP-specific IgG in most animals (Fig. 2b), suggesting the lack of a cross-protective humoral immune response, at least given the vaccine dose levels and administration regimen used here. Regardless, these data confirm the effectiveness of rVSV-SUDV immunization against GPA-SUDV challenge, demonstrating 100% protection from morbidity and mortality in the guinea pig model.

### Immunization with rVSV-EBOV provides limited cross-protection from lethal SUDV infection

To determine whether immunization with rVSV-EBOV could offer heterologous protection against SUDV challenge in the guinea pig model, 20 guinea pigs were immunized with 5 × 10^6^ TCID_50_ of rVSV-EBOV and, 28 days later, 14 animals were challenged with GPA-SUDV while the remaining 6 were challenged with GPA-EBOV (Supplementary Fig. 1c, e). Ten guinea pigs immunized with rVSV-LASV were used as controls, with 5 animals challenged with GPA-SUDV and the other 5 challenged with GPA-EBOV.

**Fig. 3:**
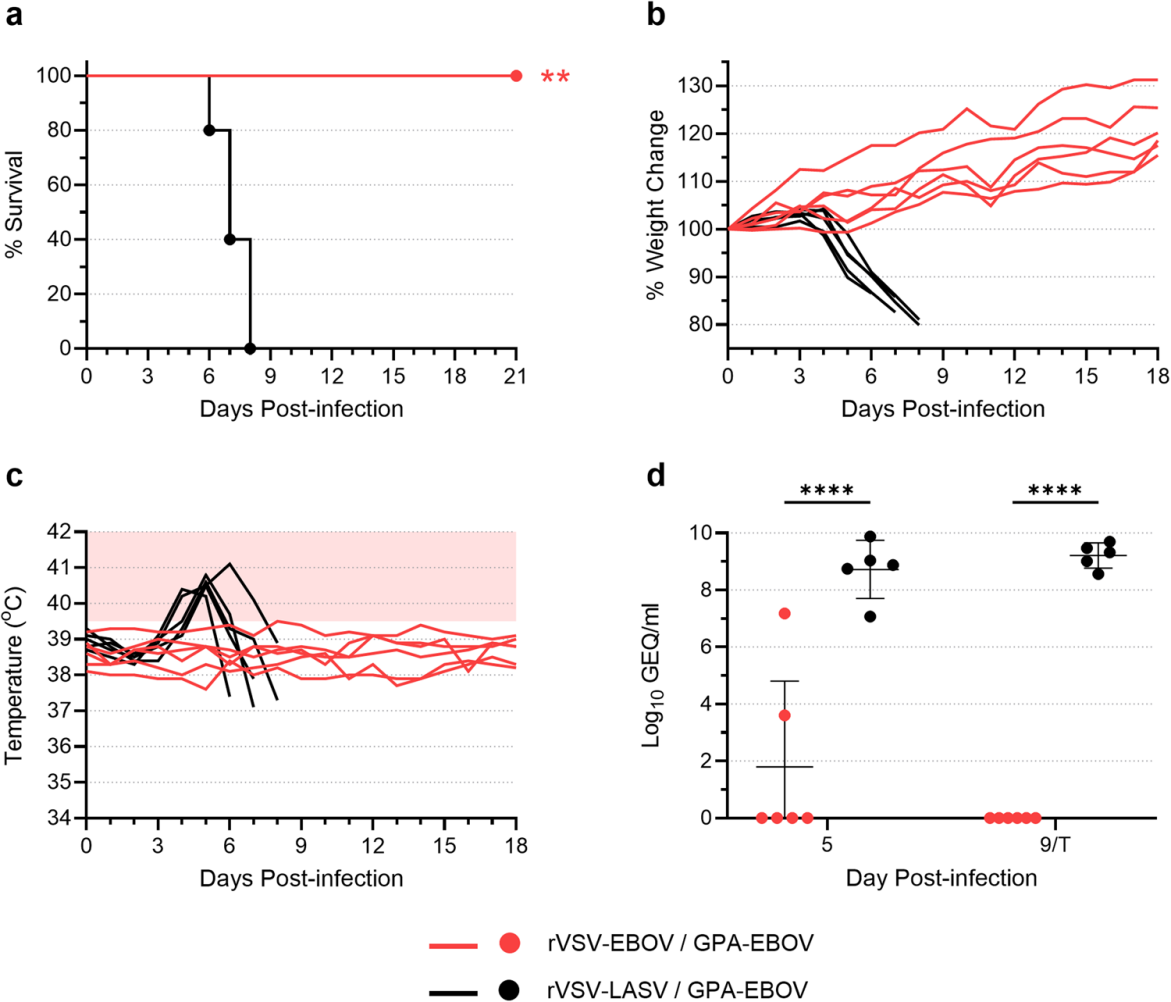
rVSV-EBOV protects against EBOV challenge in guinea pigs. Guinea pigs were vaccinated with rVSV-EBOV (*n* = 6) or rVSV-LASV (*n* = 5) at a dose of 5 × 10^6^ TCID_50_. Twenty-eight days after vaccination, animals were challenged with 1000 LD_50_ of GPA-EBOV. Animals were monitored for survival (**a**), weight change (**b**), and body temperature (**c**). The area shaded pink in (**c**) highlights temperatures above 39.5 °C. Blood samples were obtained from each animal on day 5 post-infection and either day 9 post-infection or at the terminal time point (*T*) if it occurred before day 9. Samples were assessed for levels of virus RNA via RT-qPCR, and data are presented as Log_10_ genome equivalents (GEQ) per milliliter for each animal, with means and standard deviations indicated (**d**). Survival curves (**a**) were compared using the Log-Rank test; mean virus RNA levels (**d**) were compared using a two-way ANOVA with Tukey’s multiple comparison test. **p ≤ 0.01; ****p ≤ 0.0001.

Unsurprisingly, all animals that were vaccinated with rVSV-EBOV and challenged with GPA-EBOV survived infection (Fig. 3a), showing no signs of disease, weight loss, or fever throughout the study (Fig. 3b, c). Although EBOV RNA was detected in the blood of two vaccinated animals on day 5 post-infection, no virus RNA was detectable by day 9 (Fig. 3d). In contrast, all animals vaccinated with rVSV-LASV succumbed to EBOV infection by day 8 post-infection, exhibiting dramatic weight loss, a spike in body temperatures, and very high levels of virus RNA in blood samples collected on day 5 and at the terminal time point (Fig. 3a–d). These data confirm the outstanding protective efficacy of rVSV-EBOV against EBOV challenge.

**Fig. 4:**
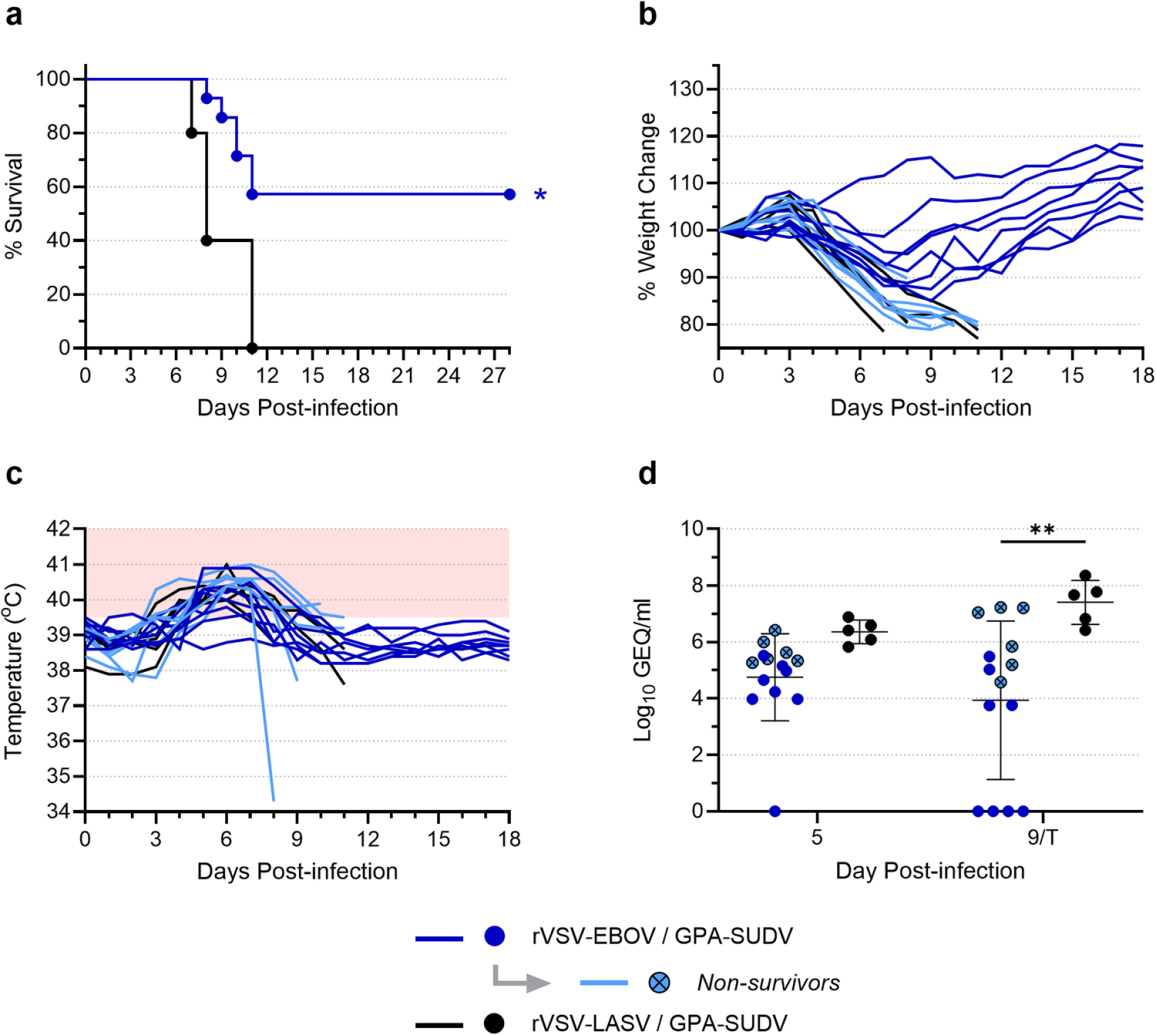
rVSV-EBOV provides limited cross-protection against SUDV challenge in guinea pigs. Guinea pigs were vaccinated with rVSV-EBOV (*n* = 14) or rVSV-LASV (*n* = 5) at a dose of 5 × 10^6^ TCID_50_. Twenty-eight days after vaccination, animals were challenged with 1000 LD_50_ of GPA-SUDV. Animals were monitored for survival (**a**), weight change (**b**), and body temperature (**c**). The area shaded pink in (**c**) highlights temperatures above 39.5 °C. Blood samples were obtained from each animal on day 5 post-infection and either day 9 post-infection or at the terminal time point (T) if it occurred before day 9. Samples were assessed for levels of virus RNA via RT-qPCR, and data are presented as Log_10_ genome equivalents (GEQ) per milliliter for each animal, with means and standard deviations indicated (**d**). Data from animals that were vaccinated with rVSV-EBOV but did not survive challenge with GPA-SUDV are highlighted in light blue and indicated with an “*x*” on the symbol. Survival curves (**a**) were compared using the Log-Rank test; mean virus RNA levels (**d**) were compared using a two-way ANOVA with Tukey’s multiple comparison test. *p ≤ 0.05; **p ≤ 0.01.

Remarkably, of the 14 guinea pigs that were vaccinated with rVSV-EBOV and challenged with GPA-SUDV, 8 animals survived (Fig. 4a; Supplementary Fig. 2). Of the surviving animals, 5 showed signs of moderate disease, with animals losing between 8 and 15% of their body weight and most exhibiting a mild to moderate fever (Fig. 4b, c; Supplementary Fig. 2). The remaining 3 survivors lost very little weight (<5%, if any) and showed no outward signs of disease, although two of them did exhibit a fever (Fig. 4b, c; Supplementary Fig. 2). In contrast, the 6 rVSV-EBOV-vaccinated animals that succumbed to SUDV all exhibited severe signs of SUDV disease, with significant weight loss and pronounced fevers (Fig. 4a–c; Supplementary Fig. 2). Likewise, the control animals that were vaccinated with rVSV-LASV and challenged with GPA-SUDV also exhibited severe signs of disease, succumbing between days 7 and 11 post-infection following significant weight loss and fever (Fig. 4a–c).

At day 5 post-infection, SUDV RNA was detected in most animals—regardless of vaccination (Fig. 4d). All but one of the 8 surviving animals showed moderate levels of virus RNA, while the 6 non-survivors all exhibited RNA levels that, on average, trended higher than that of the survivors, although the difference was not statistically significant (Supplementary Fig. 3). The rVSV-LASV-vaccinated animals had slightly higher levels of virus RNA, but this was also not statistically different compared to the rVSV-EBOV-vaccinated animals. At day 9 post-infection, four of the surviving animals had no detectable SUDV RNA, while the other four survivors showed moderate levels of RNA (Fig. 4d). All non-surviving animals had significantly higher levels of RNA than the survivors (Supplementary Fig. 3). Although the overall difference in RNA levels between the rVSV-EBOV- and rVSV-LASV-vaccinated animals was statistically significant (Fig. 4d), the difference between the non-survivors in each vaccine group was not (Supplementary Fig. 3).

**Fig. 5:**
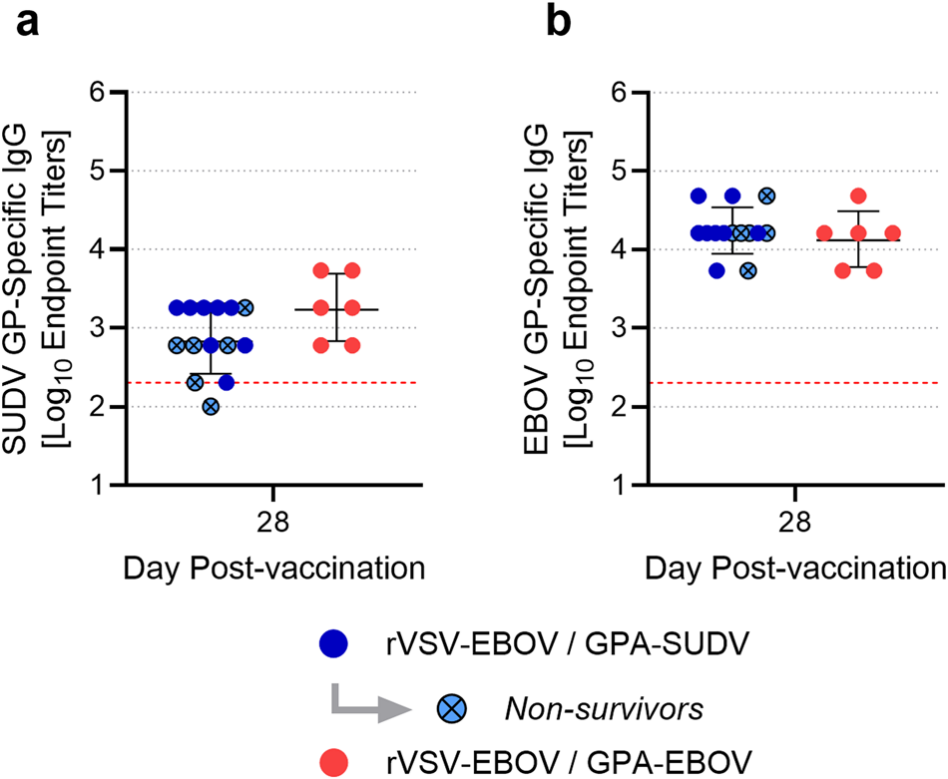
rVSV-EBOV elicits a cross-reactive humoral immune response. Serum samples were obtained from all animals 28 days after vaccination with rVSV-EBOV and prior to challenge with GPA-SUDV or GPA-EBOV. Samples were assessed for levels of SUDV GP-specific IgG (**a**) or EBOV GP-specific IgG (**b**) via ELISA. Data are presented as Log_10_ endpoint titers for each animal, with the geometric means and standard deviations indicated. The lower limit of detection is indicated with a red dashed line. Data from animals that were vaccinated with rVSV-EBOV but did not survive challenge with GPA-SUDV are highlighted in light blue and indicated with an “*x*” on the symbol. Mean IgG levels (**a**, **b**) were compared using an unpaired, two-tailed *t* test; all comparisons resulted in *p* values that were >0.05 (i.e., not significant).

Unlike the animals vaccinated with rVSV-SUDV, which did not exhibit an IgG response against EBOV GP (Fig. 2b), all rVSV-EBOV-vaccinated animals exhibited a heterologous IgG response against SUDV GP prior to challenge, albeit to a lesser degree than EBOV GP (Fig. 5). Interestingly, although the mean SUDV GP-specific IgG endpoint titer was slightly lower in the non-survivors compared to the survivors, the difference was not significant (Fig. 5a and Supplementary Fig. 4). These data suggest that other aspects of the immune response elicited by rVSV-EBOV—such as cellular immunity—may play a role in cross-protection.

### Back-challenge with EBOV but not SUDV results in lethal disease

To further investigate the degree of cross-protection elicited by vaccination and infection, we performed two back-challenge experiments (Supplementary Fig. 1b, d). The 11 guinea pigs that were vaccinated with rVSV-SUDV were back-challenged with GPA-EBOV 30 days after they were originally challenged with GPA-SUDV (Fig. 1), and the 6 guinea pigs that were vaccinated with rVSV-EBOV were back-challenged with GPA-SUDV 21 days after they were originally challenged with GPA-EBOV (Fig. 3). All animals that were back-challenged with GPA-SUDV survived (Supplementary Fig. 5a), and the majority showed no signs of illness. One of the 6 animals did exhibit weight loss and a mild fever (Supplementary Fig. 5b, c), coinciding with moderate levels of virus RNA in the blood at day 5 (Supplementary Fig. 5d), but this animal recovered completely. In contrast, 8 of the 11 animals (~73%) that were back-challenged with GPA-EBOV died after exhibiting severe disease, with the survival curves showing no significant difference from that of control animals (Supplementary Fig. 6a–c). All non-surviving animals exhibited high levels of virus RNA in the blood (Supplementary Fig. 6d). The three surviving animals remained disease-free throughout the experiment and showed no detectable virus RNA at either day 5 or day 10 post-infection (Supplementary Fig. 6a–d).

The majority of animals exhibited both SUDV and EBOV GP-specific IgG, as assessed by ELISA on serum samples obtained immediately before back challenge. rVSV-EBOV vaccination followed by GPA-EBOV challenge elicited high levels of EBOV GP-specific IgG and much lower levels of SUDV GP-specific IgG (Supplementary Fig. 7). Nevertheless, since all these animals survived back-challenge with GPA-SUDV, the humoral immune response was likely protective. Similarly, rVSV-SUDV vaccination followed by GPA-SUDV challenge elicited high levels of SUDV GP-specific IgG and much lower levels of EBOV GP-specific IgG, with one animal showing no EBOV-specific antibody activity (Supplementary Fig. 8). Interestingly, the levels of heterologous antibodies were higher than what was observed following vaccination only (c.f. Figure 2b), suggesting that challenge with GPA-SUDV boosted the immune response and enhanced cross-reactivity. However, the overall low levels of EBOV GP-specific antibodies were apparently not sufficient to protect against back-challenge with GPA-EBOV, since most animals—particularly those with the lowest antibody titers—did not survive (Supplementary Fig. 8b). It is also worth noting that the average level of antibodies detected prior to back-challenge was slightly lower than what was detected prior to the initial challenge. We suspect this difference may be a result of the gamma irradiation to which the second set of serum samples was exposed. Indeed, previous work has demonstrated that gamma irradiation can reduce the concentration of EBOV GP-specific antibodies in human serum^23^. Overall, these data further demonstrate that rVSV-EBOV vaccination (followed by EBOV challenge) confers cross-protection against SUDV; however, they also suggest that the converse scenario does not hold true. The majority of animals that were vaccinated with rVSV-SUDV and survived SUDV challenge were not able to overcome infection with GPA-EBOV, suggesting a lack of a cross-protective immune response.

## Discussion

The results presented here demonstrate that it is possible, in principle, to achieve cross-protection against challenge with SUDV following rVSV-EBOV immunization—at least in guinea pigs. Nearly 60% of the guinea pigs that were immunized with rVSV-EBOV survived challenge with SUDV 28 days later. Prior to challenge, almost all of these animals exhibited moderate levels of SUDV GP-specific IgG, in addition to high levels of EBOV GP-specific IgG. Since the humoral immune response is key to the protection offered by rVSV-EBOV^24^, it seems reasonable to assume that it was also responsible for providing partial protection against heterologous challenge with SUDV, although we cannot rule out the contribution of other arms of the immune response. Moreover, 100% of the guinea pigs that survived GPA-EBOV infection after rVSV-EBOV immunization also survived GPA-SUDV back-challenge. Despite the small number of animals, this result may suggest that the cross-protective immune response was boosted following GPA-EBOV infection.

In general, the ability of rVSV-based filovirus vaccines to elicit meaningful protection against heterologous viruses is difficult to sort out based on the handful of previously published reports. Moreover, the degree of cross-protection is likely influenced by a number of variables, including the vaccine virus and dose, the magnitude and duration of the immune response, the challenge virus, and the animal model. In rodents, for instance, Marzi et al. demonstrated that rVSV vaccines expressing TAFV or Reston virus GP provided complete cross-protection against mouse-adapted EBOV in mice but only limited cross-protection against GPA-EBOV in guinea pigs^25^. Interestingly, vaccination of mice with an rVSV vaccine expressing SUDV GP resulted in only 75% survival following EBOV challenge, while the same vaccination in guinea pigs offered no cross-protection^25^. Similarly, the results from our back-challenge experiment showed that vaccination with rVSV-SUDV followed by infection with GPA-SUDV offered only minimal protection against GPA-EBOV (Supplementary Fig. 6). These data are in contrast to the reciprocal set of experiments, in which we observed significant cross-protection against GPA-SUDV in guinea pigs immunized with rVSV-EBOV (Fig. 4) and complete cross-protection in animals that had previously survived GPA-EBOV infection following rVSV-EBOV vaccination (Supplementary Fig. 5). Thus, data from rodent experiments supports the notion that rVSV-based filovirus vaccines can elicit cross-protective immune responses; however, these data also suggest that not all rVSV vaccines and/or filovirus GPs are capable of inducing equivalent cross-reactive immune responses. Further work will therefore be necessary to understand the quality of the immune response elicited by each of these vaccines in the guinea pig model. Of particular interest is whether multiple successive—or even concurrent—doses of different rVSV vaccines might elicit a more potent and broadly cross-protective immune response. It might also be useful to gain a deeper understanding of the differences in virus pathogenicity between GPA-EBOV and GPA-SUDV, which may affect the likelihood of cross-protection.

The guinea pig models of filovirus infection have proven to be reliable and robust systems for understanding filovirus pathogenicity and, in particular, evaluating novel countermeasures. Current guinea pig models for EBOV and SUDV, as well as MARV, accurately recapitulate many of the hallmarks of filovirus disease as it is observed in NHPs and humans, and these models are routinely used to evaluate novel therapeutics and vaccines^26^. Indeed, of all the small animal models for filovirus infection, the guinea pig model is thought to have the best predictive efficacy for countermeasure evaluation^27^. Considering the additional practical advantages of this animal model—including its low cost, high degree of availability, ease of handling in high containment laboratories, and reduced ethical considerations—the guinea pig represents a critical scientific tool. Nevertheless, this animal model is not without its disadvantages. Because guinea pigs are naturally resistant to severe disease caused by wild-type filoviruses, including SUDV, the model relies on guinea pig-adapted virus strains^28^. Each guinea pig-adapted filovirus contains a different complement of genomic mutations that confer virulence in guinea pigs^29^, and whether these mutations influence the outcome of countermeasure evaluation is unknown. Work with guinea pigs also continues to be hampered by a lack of species-specific reagents—especially those for dissecting the immune response to vaccination and infection. In particular, the lack of guinea pig-specific reagents limits our ability to dissect the cellular immune response to vaccination and infection, which might have shed even more light on the results obtained in our study. Accordingly, NHPs remain the gold-standard animal model for filoviruses^30^, and the cross-protective efficacy of rVSV-EBOV should ideally be confirmed in this model.

To that end, limited results suggest that some degree of cross-protection is possible in NHPs under certain conditions. In cynomolgus macaques, 2 × 10^7^ PFU of rVSV-EBOV provided partial protection following BDBV challenge, but rVSV-TAFV did not^31^. Similarly, vaccination with 1 × 10^7^ PFU rVSV-SUDV followed by a boost with the same dose of rVSV-EBOV offered partial protection against BDBV challenge, yet a blended vaccine consisting of both rVSV-EBOV and rVSV-SUDV was not effective^32^. Conversely, a blended vaccine consisting of rVSV-EBOV, rVSV-SUDV, and rVSV-MARV was able to confer cross-protection against challenge with TAFV^22^. However, with respect to SUDV challenge, cross-protection seems more difficult to achieve. A single cynomolgus macaque immunized with 3 × 10^7^ PFU rVSV-EBOV and then challenged 28 days later with SUDV did not survive^22^, and 3 of 4 animals that had survived EBOV infection following rVSV-EBOV vaccination (1 × 10^7^ PFU) succumbed to subsequent challenge with SUDV variant Gulu^21^. Interestingly, a recent report demonstrated that immunization of cynomolgus macaques with 1 × 10^7^ PFU of rVSV-EBOV followed by infection with EBOV resulted in a SUDV GP-specific IgG response that persisted, albeit at low levels, up to 290 days post-vaccination^33^. However, following a control immunization with rVSV-MARV, these animals were not protected from subsequent challenges with SUDV, with 4 of 5 succumbing to the disease. Together, these data suggest that rVSV-EBOV does not reliably generate an immune response in NHPs that is sufficiently cross-protective against SUDV. How, then, do we reconcile these data with our observations of cross-protection in guinea pigs immunized with rVSV-EBOV? While the guinea pig model, in general, is considered more stringent than other rodent models, it may be less stringent than the NHP model, offering a lower bar for protection than is required in monkeys. Indeed, as alluded to above, we cannot exclude the possibility that differences in the pathogenic processes related to the use of GPA-SUDV could have influenced the outcome of vaccination or challenge. Differences in vaccine dose and the time between vaccination and challenge may have also contributed to the discordant results in guinea pigs and NHPs. In our study, guinea pigs were immunized with 5 × 10^6^ TCID_50_ rVSV-EBOV, while the NHPs in the study by Marzi et al. were immunized with 1 × 10^7^ PFU^33^. The degree to which these different doses might have affected the outcome is unclear. Likewise, the relatively short duration between rVSV-EBOV vaccination and challenge in our experiments (28 days) versus those by Marzi et al. (318 days) likely also played a role^33^. As vaccine-elicited antibody titers are expected to decrease over time, it is unclear to what degree rVSV-EBOV would continue to offer protection in guinea pigs against SUDV beyond 28 days post-vaccination. Additionally, little is known about how the immune responses to these vaccines compare across different species, which means we cannot rule out the possibility that such differences could have contributed to the cross-protection observed in guinea pigs and the lack of cross-protection observed in NHPs. Clearly, there are many questions left to answer regarding the cross-protective potential of rVSV-EBOV, which further underscores the need for additional research into the immune responses elicited by filovirus vaccines in both guinea pigs and NHPs.

Whether or not rVSV-EBOV is capable of eliciting meaningful cross-protection against SUDV, it is worth remembering that rVSV-SUDV is already known to offer robust protection against SUDV. Our data demonstrate that rVSV-SUDV elicits a uniformly high SUDV GP-specific antibody response in guinea pigs as early as 14 days after a single administration and even when given at a relatively low dose of 2 × 10^3^ PFU. These data confirm that the rVSV-SUDV vaccine is highly effective at preventing Sudan virus disease. Additionally, Marzi et al.^33^ have recently demonstrated the effectiveness of rVSV-SUDV in NHPs, and a past report showed that the vaccine also works as a post-exposure treatment^34^. Given the success of the rVSV-EBOV vaccine, and abundant data demonstrating the efficacy of analogous VSV vaccines for other filoviruses, it is long past time for rVSV-SUDV to be advanced to clinical trials.

## Methods

### Animal ethics and biosafety statement

All animal experiment protocols were reviewed and approved by the Animal Care Committee at the Canadian Science Centre for Human and Animal Health (CSCHAH), Winnipeg, Manitoba, in accordance with guidelines from the Canadian Council on Animal Care (CCAC). All staff working on animal experiments completed education and training programs according to the standard protocols appropriate for this level of biosafety. All work with infectious SUDV and EBOV was performed in the containment level (CL)-4 laboratories at the CSCHAH in accordance with standard operating protocols.

### Viruses

Guinea pig-adapted Sudan virus variant Boneface (GPA-SUDV; Sudan virus/NML/C.porcellus-lab/SSD/1976/Nzara-Boneface-GP; Genbank accession number KT750754.1)^29^ and guinea pig-adapted Ebola virus variant Mayinga (GPA-EBOV)^35^ were used as challenge viruses in this study. Recombinant vesicular stomatitis virus (rVSV)-based vaccines expressing SUDV GP, EBOV GP, or LASV G in place of VSV G were generated previously using standard reverse genetics procedures^9^. Briefly, VSV G was deleted from a plasmid encoding the full-length genome of the vesicular stomatitis Indiana virus and replaced with the coding sequence for SUDV GP, EBOV GP, or LASV GP. Recombinant viruses were rescued by transfecting BHK-T7 cells with the altered full-length genome plasmid as well as helper plasmids encoding VSV N, P, and L. Supernatants from the transfected cells were blind passaged on Vero E6 cells (P1) and used to generate a P2 stock. All virus was sequence confirmed and stored at −80°C until use.

### rVSV-SUDV efficacy against GPA-SUDV in guinea pigs

To evaluate the protective efficacy of rVSV-SUDV immunization against GPA-SUDV challenge in female Hartley guinea pigs (Charles River Laboratories), groups of 6 animals each were immunized via the intramuscular route with either 2 × 10^5^ PFU or 2 × 10^3^ PFU of rVSV-SUDV (Supplementary Fig. 1a). We chose the highest dose level possible based on the titer of the vaccine virus and then chose a second dose level two logs lower in an attempt to identify a dose-response effect. A control group of six animals received an equivalent volume of 0.9% saline. Twenty-eight (28) days post-vaccination, all animals were inoculated with 1000 times the median lethal dose (LD_50_) of GPA-SUDV via intraperitoneal (IP) injection. Animals were monitored for disease and survival up to 30 days post-infection. Weights were recorded daily for all animals up to day 16, as were body temperatures (as measured via subcutaneously implanted transponders). EDTA blood and/or serum samples were obtained from all animals prior to vaccination, on day 5 post-infection, and on either day 9 post-infection or at the animal’s terminal time point if it occurred before day 9.

On day 30 post-infection, all immunized animals (*n* = 11) were back-challenged with 1000 LD_50_ of GPA-EBOV via IP injection (Supplementary Fig. 1b). EDTA blood and/or serum samples were obtained prior to back-challenge, on day 5 post-infection, and on either day 10 post-infection or at each animal’s terminal time point if it occurred before day 10. Animals were monitored for disease and survival up to 21 days post-infection with weights and temperatures recorded daily up to day 16.

### rVSV-EBOV efficacy against GPA-SUDV in guinea pigs

To evaluate the cross-protective efficacy of rVSV-EBOV immunization against GPA-SUDV challenge in female Hartley guinea pigs (Charles River Laboratories), 20 animals were immunized via the IP route with 5 × 10^6^ TCID_50_ (roughly equivalent to 1 × 10^7^ PFU) of rVSV-EBOV (Supplementary Fig. 1c, e). A control group of 10 animals were immunized via the IP route with 5 × 10^6^ TCID_50_ of rVSV-LASV. Twenty-eight (28) days post-vaccination, 14 of the animals that had been immunized with rVSV-EBOV were challenged with 1000 LD_50_ of GPA-SUDV via the IP route, while the remaining six were challenged with 1000 LD_50_ of GPA-EBOV. At the same time point, 5 of the control animals (immunized with rVSV-LASV) were challenged with 1000 LD_50_ of GPA-SUDV, and the other 5 were challenged with 1000 LD_50_ of GPA-EBOV. Animals challenged with GPA-EBOV were monitored for disease and survival up to day 21 post-infection, while animals challenged with GPA-SUDV were monitored for disease and survival up to day 28. Weights were recorded daily for all animals up to day 18, as were body temperatures (as measured via subcutaneously implanted transponders). EDTA blood and/or serum samples were obtained from all animals prior to vaccination, on day 5 post-infection, and on either day 9 post-infection or at the animal’s terminal time point, if it occurred before day 9.

On day 21 post-infection, all animals (*n* = 6) that had been immunized with rVSV-EBOV and challenged with GPA-EBOV were back-challenged with 1000 LD_50_ of GPA-SUDV via IP injection (Supplementary Fig. 1d). EDTA blood and/or serum samples were obtained prior to back-challenge and on days 5 and 10 post-infection. Animals were monitored for disease and survival up to 21 days post infection with weights and temperatures recorded daily up to day 16.

### Virus RNA quantification

EDTA blood samples were inactivated using Buffer AVL (Qiagen) and ethanol, according to the manufacturer’s instructions. Viral RNA was extracted from these samples using the KingFisher Viral NA Kit (Thermo Fisher) on the KingFisher Apex per the manufacturer’s protocol. GPA-SUDV and GPA-EBOV RNA levels were determined by reverse transcription quantitative PCR (RT-qPCR) using the TaqPath 1-Step Multiplex Master Mix (Thermo Fisher) on the Applied Biosystems QuantStudio 3, along with the SUDV-specific primers (forward, 5′- CAGAAGACAATGCAGCCAGA-3′; reverse, 5′- TTGAGGAATATCCCACAGGC-3′; probe, 5′-6-FAM-CTGCTAGCT/Zen/TGGCCAAAGTCACAAG-IABkFQ-3′) or EBOV-specific primers (forward, 5′- CAGCCAGCAATTTCTTCCAT-3′; reverse, 5′- TTTCGGTTGCTGTTTCTGTG-3′; probe 5′-6-FAM-ATCATTGGCGTACTGGAGGAGCAG-IABkFQ-3′). Cycling conditions were as follows: 25 °C for 2 min, 53 °C for 10 min, and 95 °C for 2 min, followed by 40 cycles of 95 °C for 3 s and 60 °C for 30 s. Standard curves were generated from plasmids encoding SUDV L or EBOV L and were used to convert the cycle threshold (Ct) values to genome equivalents per milliliter (GEQ/mL).

### IgG ELISAs

SUDV GP-specific IgG and EBOV-specific IgG levels were quantified in serum samples by indirect ELISA. Half-area high-binding 96-well assay plates (Corning) were coated using transmembrane domain-deleted SUDV GP or EBOV GP proteins (IBT Bioservices) prepared in pH 9.5 carbonate buffer in a 30-µl volume (1 µg/ml) at 4 °C for overnight. On the day of the experiment, after removing the coating solution, plates were incubated with 100 µl of 5% skim milk (BD Biosciences) prepared in 0.1% Tween-20 in PBS for 3 h at 37 °C. Serial dilutions of serum samples prepared in 2% milk were then applied to the plates (30 µl/well) and allowed to incubate at 4 °C overnight. Following four washes with 0.1% Tween-20/PBS, the plates were incubated for 1 h at 37 °C with an HRP-conjugated goat anti-guinea pig IgG(H + L) secondary antibody (KPL; Catalog No. 5220-0366) diluted 1:5000 in 2% milk, followed by four washes with 0.1% Tween-20/PBS. The plates were then incubated in a TMB solution (Life Technologies) for ~30 min in darkness before optical density (OD) signals were measured at 650 nm using a Synergy HTX plate reader (Biotek). Endpoint dilution titers were calculated by determining the highest dilution that gave an average OD 650 reading greater than or equal to the cut-off OD value, which was set as the mean OD value for pre-vaccine serum samples plus three times the standard deviation. When the endpoint titer was determined to lie below the lower limit of detection, an arbitrary value of 1:100 was assigned.

### Statistical analyses

GraphPad Prism version 9 was used to perform all statistical tests and generate all graphs. The Kaplan–Meier survival curves were compared using the Log-Rank test with the Bonferroni correction for multiple comparisons (for Fig. 1a and Supplementary Fig. 6a). The two-way ANOVA test, along with Tukey’s multiple comparison tests, was used to compare the means in Figs. 1d, 2, 3d, and 4d, as well as Supplementary Figs. 3, 5d, and 6d with 95% confidence intervals. An unpaired, two-tailed *t* test was used to compare the means in Fig. 5 and Supplementary Fig. 4 with a 95% confidence interval. Statistically significant differences are indicated with asterisks, where a *P* value ≤0.05 was marked with one asterisk (*), ≤0.01 was marked with two asterisks (**), ≤0.001 was marked with three asterisks (***), and ≤0.0001 was marked with four asterisks (****). In instances where the Bonferroni correction or Tukey’s multiple comparison tests were performed, corrected *P* values were used.

### Reporting summary

Further information on research design is available in the Nature Research Reporting Summary linked to this article.

## Supplementary information

Supplemental Figures

REPORTING SUMMARY

## Data Availability

Data supporting the conclusions of this study can be found in this article or the Supplementary information. Any additional data are available from the corresponding author upon reasonable request.
