# Supplementary material for: The rVSV-EBOV vaccine provides limited cross-protection against Sudan virus in guinea pigs
Source: NPJ Vaccines. 2023 Jun 10;8:91. doi: 10.1038/s41541-023-00685-z (PMC10257645; doi:10.1038/s41541-023-00685-z)
Supplement: Supplementary file 1 — Supplemental Figures [file 41541_2023_685_MOESM1_ESM.pdf]

## SUPPLEMENTARY MATERIALS

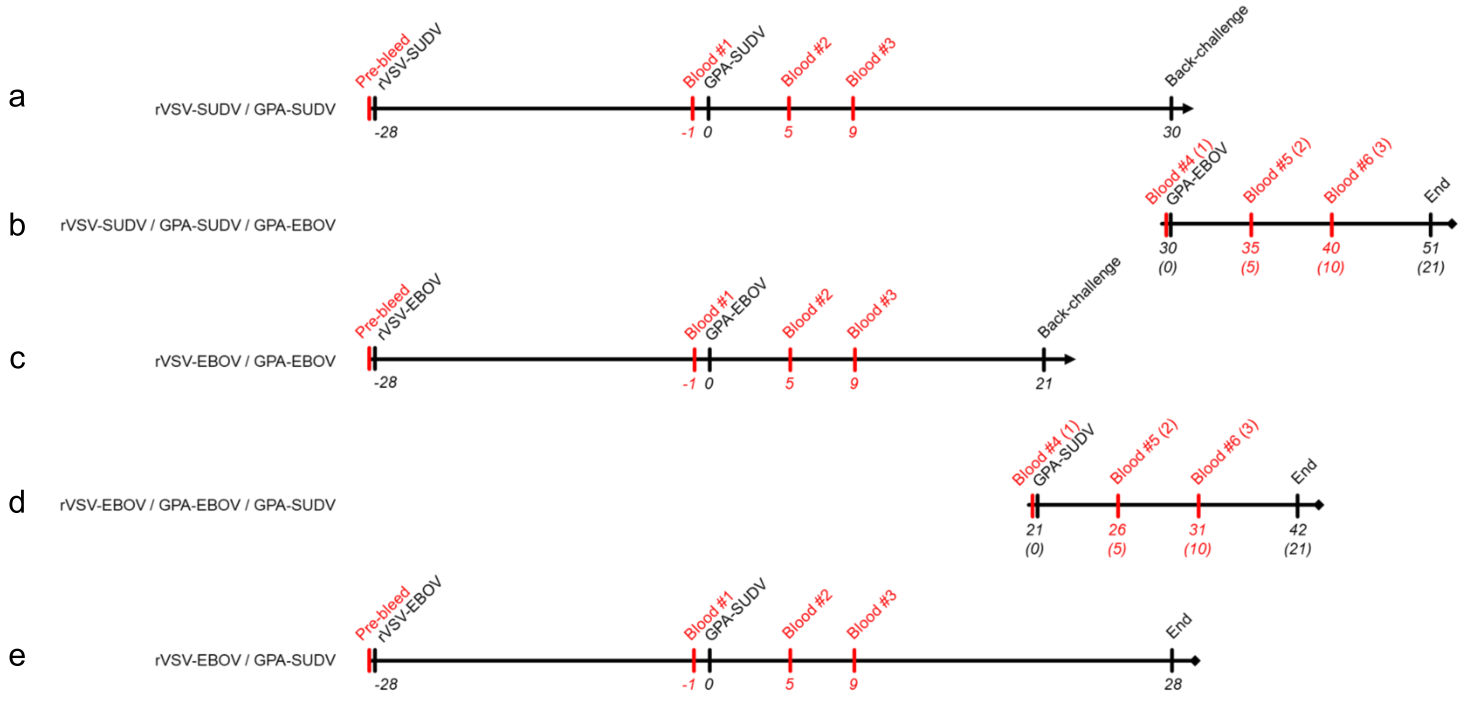


**Supplementary Fig. 1. Study Outline.** A graphical depiction of the experimental timelines for each challenge experiment. (a) To assess rVSV-SUDV protection against GPA-SUDV challenge, guinea pigs were immunized with 2 x $10^5$ PFU or 2 x $10^3$ PFU rVSV-SUDV and then challenged with 1000 $LD_{50}$ GPA-SUDV 28 days later. (b) On day 30 post-infection, the surviving animals from (a) were back-challenged with 1000 $LD_{50}$ of GPA-EBOV to assess cross-protection. (c) To assess rVSV-EBOV protection against GPA-EBOV challenge, guinea pigs were immunized with 5 x $10^6$ $TCID_{50}$ rVSV-EBOV and then challenged with 1000 $LD_{50}$ GPA-EBOV 28 days later. (d) On day 21 post-infection, the surviving animals from (c) were back-challenged with 1000 $LD_{50}$ of GPA-SUDV to assess cross-protection. (e) To assess rVSV-EBOV cross-protection against GPA-SUDV challenge, guinea pigs were immunized with 5 x $10^6$ $TCID_{50}$ rVSV-EBOV and then challenged with 1000 $LD_{50}$ GPA-SUDV 28 days later. The experiment was terminated on day 28 post-infection. In general, blood samples were obtained from all animals before vaccination (Pre-bleed), prior to challenge (Blood #1, day -1), and again on days 5 (Blood #2) and 9 or 10 (Blood #3). Blood samples (Terminal) were also obtained whenever an animal met the humane criteria for euthanasia.

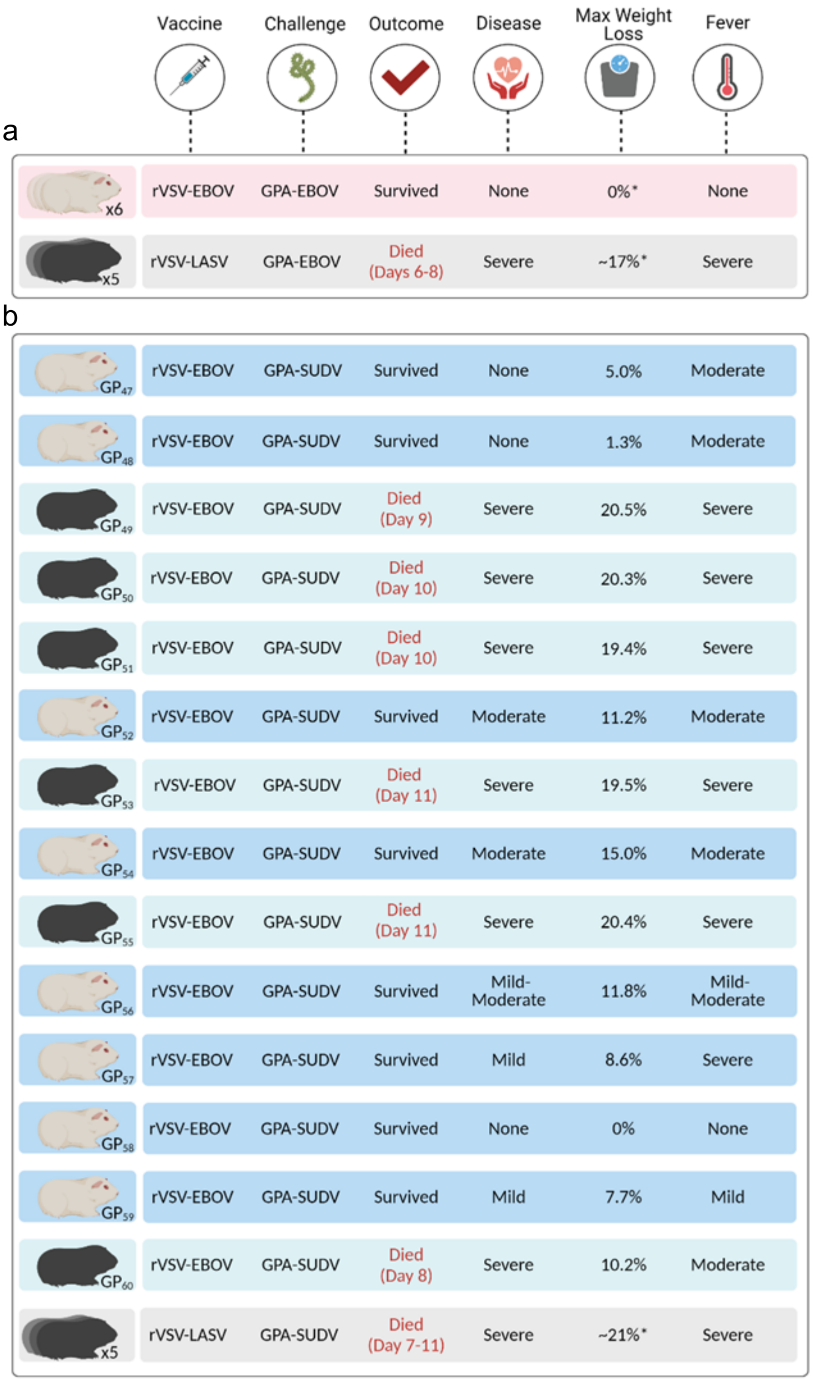


**Supplementary Fig. 2. Summary of cross-protection in guinea pigs vaccinated with rVSV-EBOV.** The diagram depicts a qualitative summary of the disease observed in guinea pigs vaccinated with rVSV-EBOV or rVSV-LASV and challenged with GPA-EBOV (a) or GPA-SUDV (b). A summary is provided for each of the 14 animals (ID numbers indicated) that were vaccinated with rVSV-EBOV and challenged with GPA-SUDV, while a collective summary is provided for all other animals. Disease severity was qualitatively categorized as severe, moderate, or mild based on the observation of clinical

signs, including animal activity and posture, as well as weight loss and body temperature. Fever was categorized based on maximum recorded temperature: >40.5ºC, severe; >40ºC, moderate; >39.5ºC mild. *, indicates an average value. This figure was created with BioRender.com.

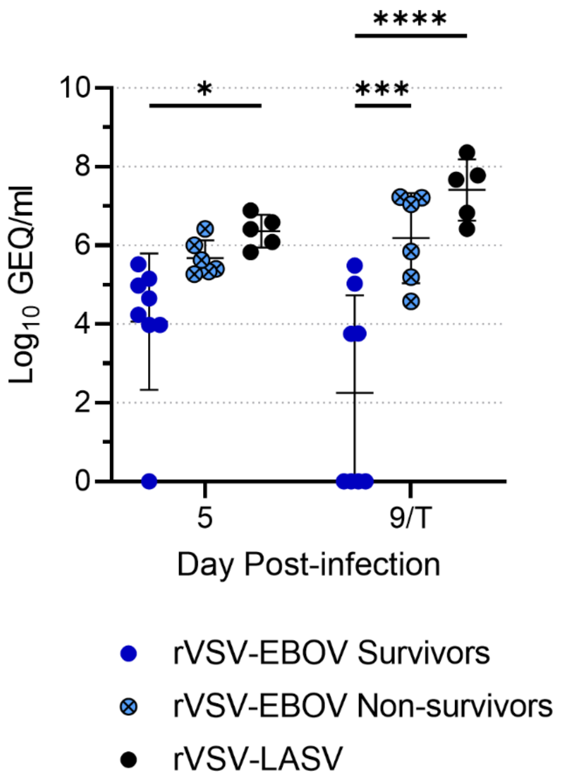


**Supplementary Fig. 3. Statistical analysis of virus RNA levels in guinea pigs vaccinated with rVSV-EBOV and challenged with GPA-SUDV.** To determine whether the levels of virus RNA were significantly different between the rVSV-EBOV-vaccinated survivors and non-survivors, data from Fig. 4d was re-plotted to separate the rVSV-EBOV vaccinated survivors from the non-survivors. A two-way ANOVA test, along with Tukey's multiple comparison test, was performed. At day 5, there was no significant difference between the virus RNA levels in rVSV-EBOV vaccinated survivors and non-survivors, although the level of virus RNA in the rVSV-LASV-vaccinated control animals was significantly higher than in the rVSV-EBOV vaccinated survivors. At day 9 or the terminal time point, the average level of virus RNA in the non-survivors was significantly greater than that in the survivors but not significantly different from that in the control animals. Data from animals that were vaccinated with rVSV-EBOV but did not survive challenge with GPA-SUDV are highlighted in light blue and indicated with an "x" on the symbol. *, $p \leq 0.05$; ***, $p \leq 0.001$; ****, $p \leq 0.0001$.

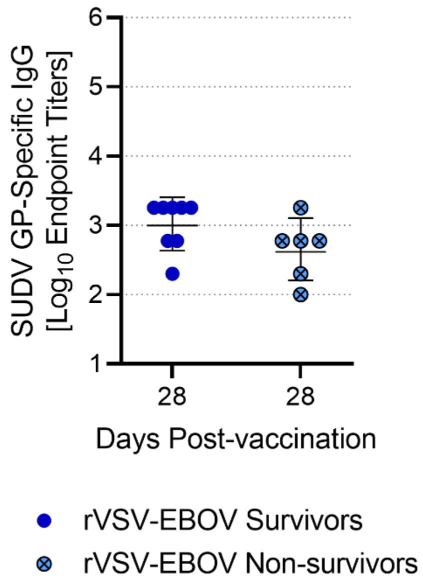


**Supplementary Fig. 4. Statistical analysis of mean SUDV GP-specific IgG levels in guinea pigs vaccinated with rVSV-EBOV and challenged with GPA-SUDV.** To determine whether the SUDV GP-specific IgG levels were significantly different between the rVSV-EBOV-vaccinated and GPA-SUDV challenged survivors and non-survivors, data from Fig. 5a was re-plotted to separate the survivors from the non-survivors. Data from animals that were vaccinated with rVSV-EBOV but did not survive challenge with GPA-SUDV are highlighted in light blue and indicated with an “x” on the symbol. An unpaired, two-tailed t-test was performed, and no significant differences were identified between the two groups.

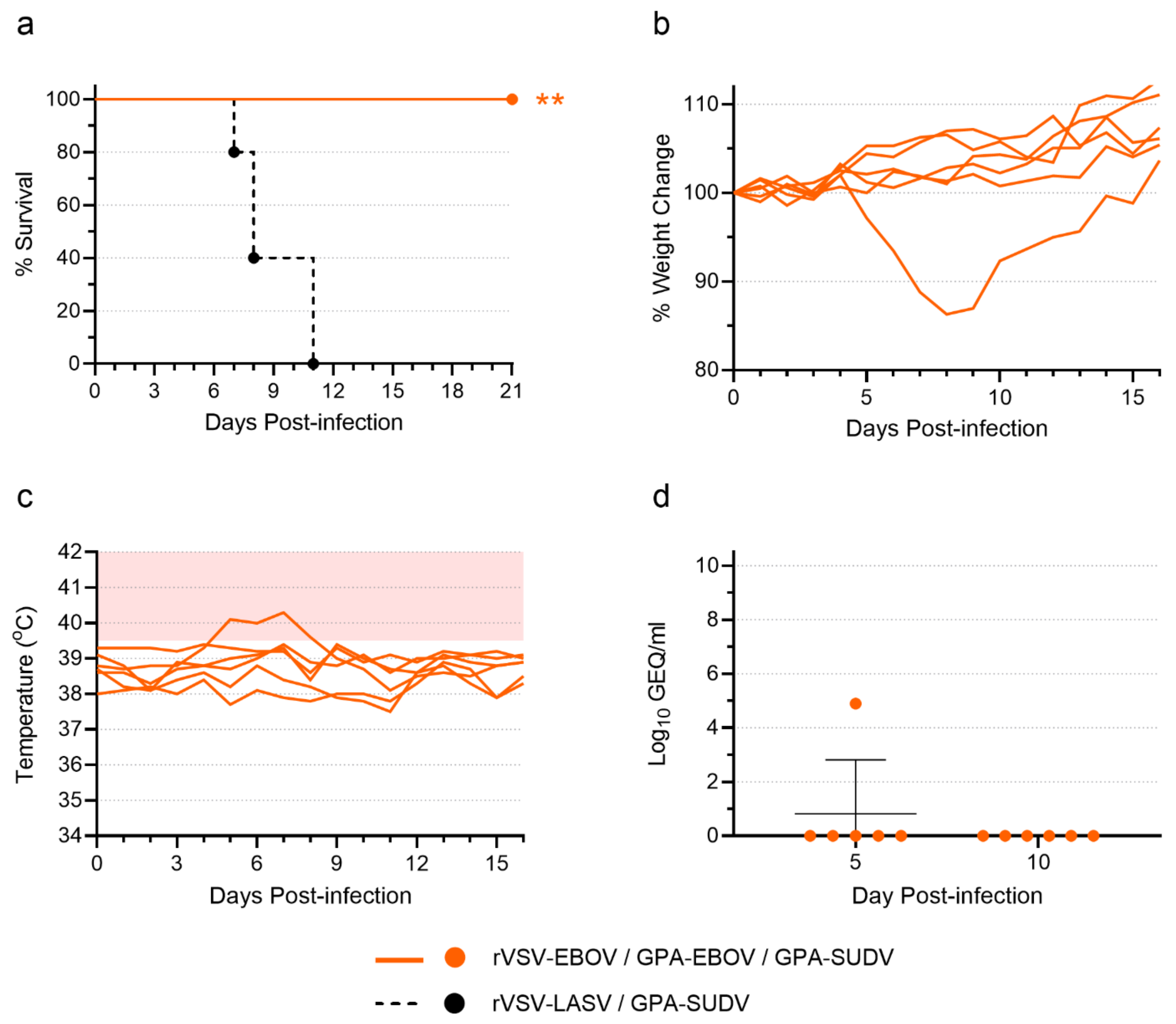


**Supplementary Fig. 5. rVSV-EBOV-vaccinated survivors from EBOV challenge are protected against SUDV back-challenge.** As described for Fig. 3, guinea pigs were vaccinated with rVSV-EBOV (n = 6) and then challenged with 1000 $LD_{50}$ GPA-EBOV. All animals survived GPA-EBOV challenge and, 21 days later, were back-challenged with 1000 $LD_{50}$ of GPA-SUDV. Animals were monitored for survival (a), weight change (b), and body temperature (c). The area shaded pink in (c) highlights temperatures above 39.5 °C. For comparison, the survival curve from animals vaccinated with rVSV-LASV and challenged with GPA-SUDV (Fig. 4) is shown as a dotted line (a). Blood samples were obtained from each animal on day 5 and 10 post-infection. Samples were assessed for levels of virus RNA via RT-qPCR, and data are presented as $Log_{10}$ genome equivalents (GEQ) per milliliter for each animal, with means and standard deviations indicated (d). Survival curves (a) were compared using the Log-Rank test; mean virus RNA levels (d) were compared using a two-way ANOVA with Tukey's multiple comparison test. **, $p \leq 0.01$.

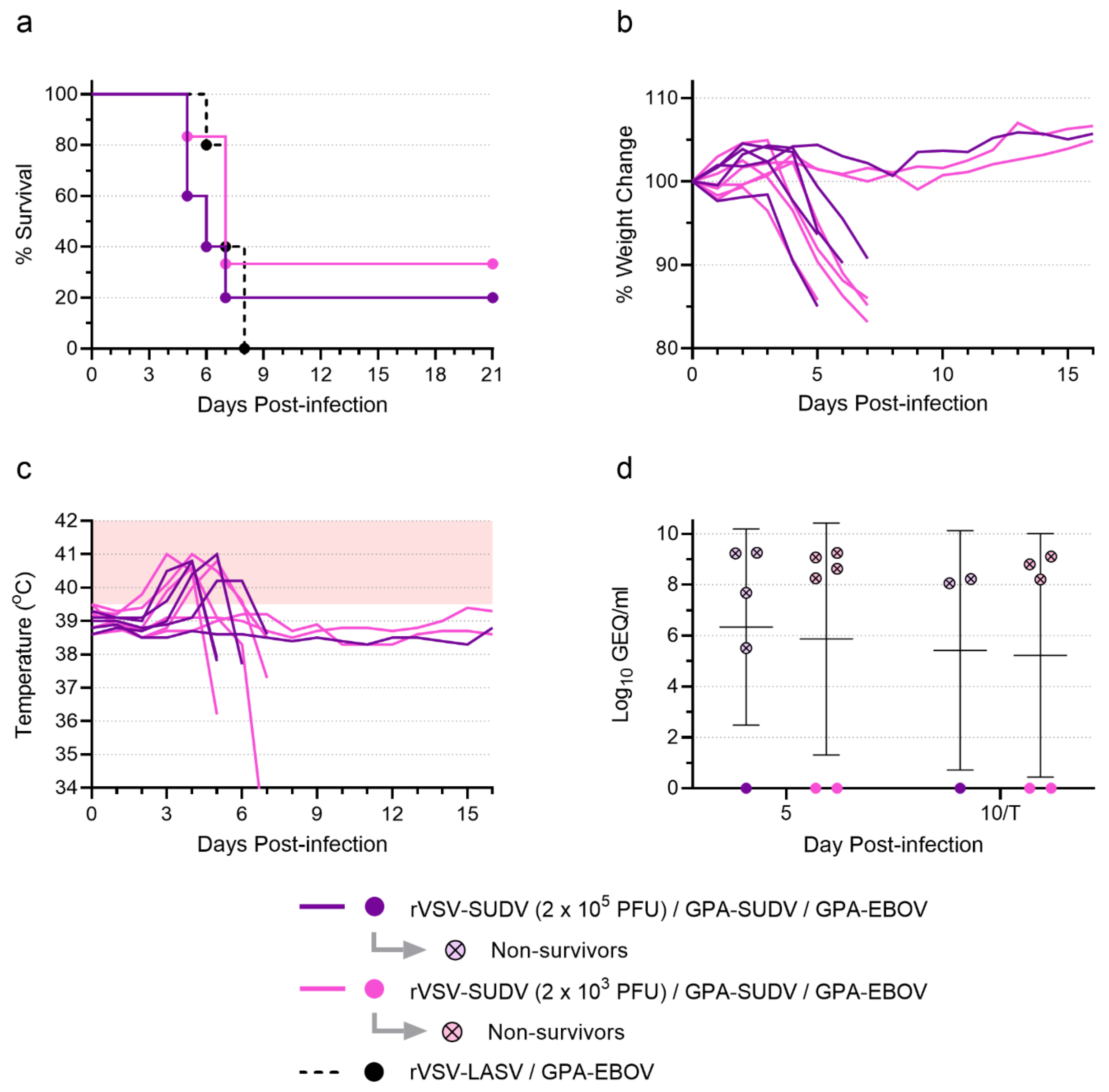


**Supplementary Fig. 6. rVSV-SUDV vaccinated survivors from SUDV challenge are not protected against EBOV back-challenge.** As described for Fig. 1, guinea pigs were vaccinated with rVSV-SUDV at a dose of either 2 x $10^5$ PFU (n = 6) or 2 x $10^3$ PFU (n = 6) and then challenged with 1000 $LD_{50}$ of GPA-SUDV. All animals survived GPA-SUDV challenge and, 30 days later, were back-challenged with 1000 $LD_{50}$ of GPA-EBOV. Note that a single vaccinated animal died during sampling in the original experiment, so the rVSV-SUDV (2 x $10^5$ PFU) group contained only 5 animals for the back challenge. Animals were monitored for survival (a), weight change (b), and body temperature (c). The area shaded pink in (c) highlights temperatures above 39.5 °C. For comparison, the survival curve from animals vaccinated with rVSV-LASV and challenged with GPA-EBOV (Fig. 3) is shown as a dotted line (a). Blood samples were obtained from each animal on day 5 post-infection and either day 10 post-infection

or at the terminal time point (T) if it occurred before day 10. Samples were assessed for levels of virus RNA via RT-qPCR, and data are presented as $Log_{10}$ genome equivalents (GEQ) per milliliter for each animal, with means and standard deviations indicated (d). Data from animals that were back-challenged with GPA-EBOV but did not survive are highlighted in light purple or light pink and indicated with an “x” on the symbol. Survival curves (a) were compared using the Log-Rank test; mean virus RNA levels (d) were compared using a two-way ANOVA with Tukey’s multiple comparison test.

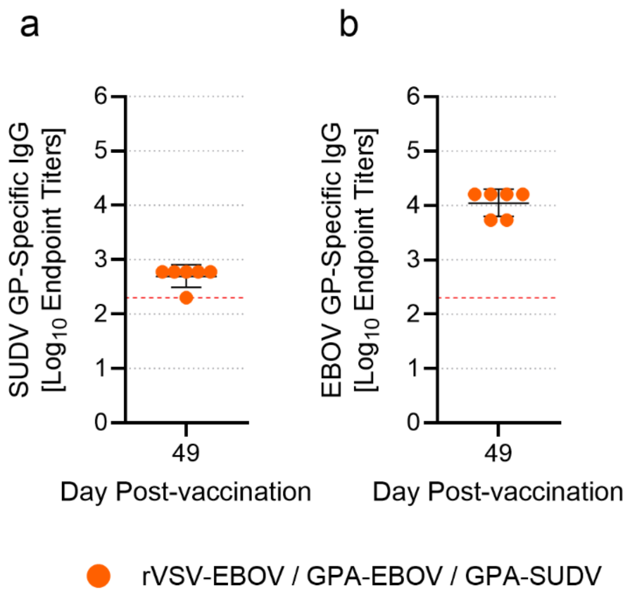


**Supplementary Fig. 7. A cross-reactive humoral immune response in animals vaccinated with rVSV-EBOV and challenged with GPA-EBOV.** As described for Fig. 3, guinea pigs were vaccinated with rVSV-EBOV (n = 6) and then challenged with 1000 $LD_{50}$ GPA-EBOV. All animals survived GPA-EBOV challenge and, 21 days later, were back-challenged with 1000 $LD_{50}$ of GPA-SUDV. Serum samples were obtained from the animals prior to back-challenge with GPA-SUDV (day 21 after GPA-EBOV challenge and day 49 after vaccination). Samples were gamma irradiated and then assessed for levels of SUDV GP-specific IgG (a) or EBOV GP-specific IgG (b) via ELISA. Data are presented as $Log_{10}$ endpoint titers for each animal, with the geometric means and standard deviations indicated. The lower limit of detection is indicated with a red dashed line.

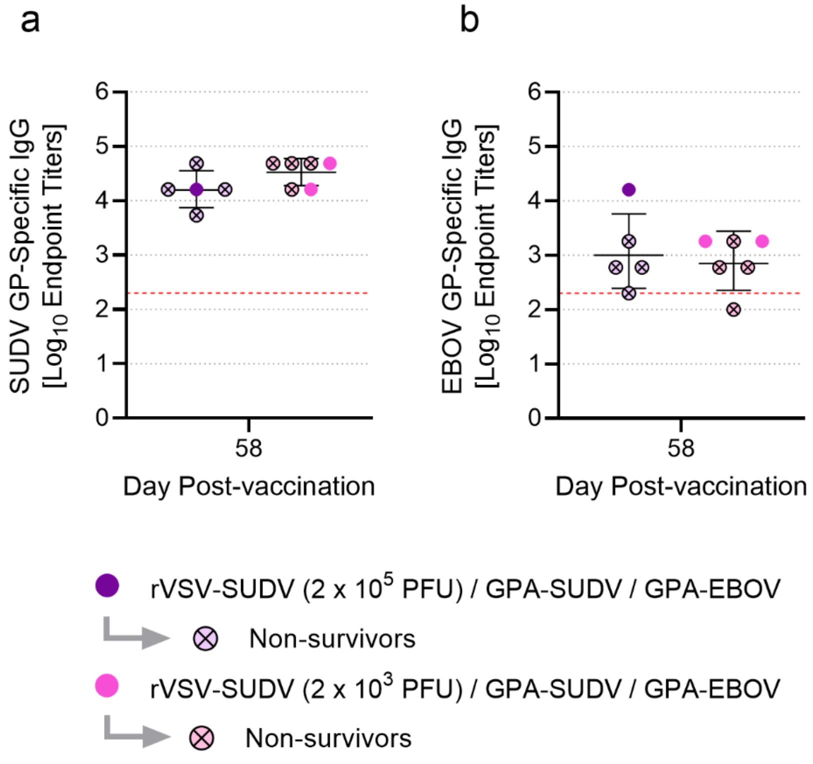


**Supplementary Fig. 8. A cross-reactive humoral immune response in animals vaccinated with rVSV-SUDV and challenged with GPA-SUDV.** As described for Fig. 1, guinea pigs were vaccinated with rVSV-SUDV at a dose of either 2 x $10^5$ PFU (n = 6) or 2 x $10^3$ PFU (n = 6) and then challenged with 1000 $LD_{50}$ of GPA-SUDV. All animals survived GPA-SUDV challenge and, 30 days later, were back-challenged with 1000 $LD_{50}$ of GPA-EBOV. Note that a single vaccinated animal died during sampling in the original experiment, so the rVSV-SUDV (2 x $10^5$ PFU) group contained only 5 animals for the back challenge. Samples were gamma irradiated and then assessed for levels of SUDV GP-specific IgG (a) or EBOV GP-specific IgG (b) via ELISA. Data are presented as $Log_{10}$ endpoint titers for each animal, with the geometric means and standard deviations indicated. The lower limit of detection is indicated with a red dashed line. Data from animals that were back-challenged with GPA-EBOV but did not survive are highlighted in light purple or light pink and indicated with an "x" on the symbol.
