# Supplementary material for: The rVSV-EBOV vaccine provides limited cross-protection against Sudan virus in guinea pigs
Source: NPJ Vaccines. 2023 Jun 10;8:91. doi: 10.1038/s41541-023-00685-z (PMC10257645; doi:10.1038/s41541-023-00685-z)
Supplement: Supplementary file 2 — REPORTING SUMMARY [file 41541_2023_685_MOESM2_ESM.pdf]

nature portfolio

Corresponding author(s): Logan Banadyga

Last updated by author(s): Apr 23, 2023

Reporting Summary

Nature Portfolio wishes to improve the reproducibility of the work that we publish. This form provides structure for consistency and transparency in reporting. For further information on Nature Portfolio policies, see our Editorial Policies and the Editorial Policy Checklist.

Statistics

For all statistical analyses, confirm that the following items are present in the figure legend, table legend, main text, or Methods section.

n/a	Confirmed	
☐	☒	The exact sample size (*n*) for each experimental group/condition, given as a discrete number and unit of measurement
☐	☒	A statement on whether measurements were taken from distinct samples or whether the same sample was measured repeatedly
☐	☒	The statistical test(s) used AND whether they are one- or two-sided<br>*Only common tests should be described solely by name; describe more complex techniques in the Methods section.*
☒	☐	A description of all covariates tested
☐	☒	A description of any assumptions or corrections, such as tests of normality and adjustment for multiple comparisons
☐	☒	A full description of the statistical parameters including central tendency (e.g. means) or other basic estimates (e.g. regression coefficient) AND variation (e.g. standard deviation) or associated estimates of uncertainty (e.g. confidence intervals)
☐	☒	For null hypothesis testing, the test statistic (e.g. *F*, *t*, *r*) with confidence intervals, effect sizes, degrees of freedom and *P* value noted<br>*Give P values as exact values whenever suitable.*
☒	☐	For Bayesian analysis, information on the choice of priors and Markov chain Monte Carlo settings
☒	☐	For hierarchical and complex designs, identification of the appropriate level for tests and full reporting of outcomes
☒	☐	Estimates of effect sizes (e.g. Cohen's *d*, Pearson's *r*), indicating how they were calculated

Our web collection on statistics for biologists contains articles on many of the points above.

Software and code

Policy information about availability of computer code

Data collection	No software was used to collect the data for this study
Data analysis	GraphPad Prism version 9 for Windows was used to perform all statistical tests and generate all graphs for this study.

For manuscripts utilizing custom algorithms or software that are central to the research but not yet described in published literature, software must be made available to editors and reviewers. We strongly encourage code deposition in a community repository (e.g. GitHub). See the Nature Portfolio guidelines for submitting code & software for further information.

Data

Policy information about availability of data

All manuscripts must include a data availability statement. This statement should provide the following information, where applicable:

- Accession codes, unique identifiers, or web links for publicly available datasets
- A description of any restrictions on data availability
- For clinical datasets or third party data, please ensure that the statement adheres to our policy

Data supporting the conclusions of this study can be found in this article or the Supplementary information. Any additional data are available from the corresponding author upon reasonable request.

Research involving human participants, their data, or biological material

Policy information about studies with human participants or human data. See also policy information about sex, gender (identity/presentation), and sexual orientation and race, ethnicity and racism.

Reporting on sex and gender	This study did not involve human participants.
Reporting on race, ethnicity, or other socially relevant groupings	This study did not involve human participants.
Population characteristics	This study did not involve human participants.
Recruitment	This study did not involve human participants.
Ethics oversight	This study did not involve human participants.

Note that full information on the approval of the study protocol must also be provided in the manuscript.

Field-specific reporting

Please select the one below that is the best fit for your research. If you are not sure, read the appropriate sections before making your selection.

☒ Life sciences ☐ Behavioural & social sciences ☐ Ecological, evolutionary & environmental sciences

For a reference copy of the document with all sections, see nature.com/documents/nr-reporting-summary-flat.pdf

Life sciences study design

All studies must disclose on these points even when the disclosure is negative.

Sample size	All animal groups in this study consisted of 5 or more animals, with the majority of groups consisting of six animals. A minimum of five animals is sufficient to confirm a difference in survival of at least 80% at the 95% confidence interval with 80% power, using standard power analysis calculations.
Data exclusions	No data were excluded from the analyses.
Replication	Each group consisted of a minimum of 5 animals, with the majority of groups consisting of at least 6 animals. Within each group, each animal served as a single replicate and multiple analyses (i.e., virus RNA quantification and IgG ELISAs) were employed to support the survival outcome of each study. Additionally, back-challenges of various vaccinated and challenged groups provided further evidence of vaccine cross-protection (or lack thereof).
Randomization	Animals were randomly assigned to each group.
Blinding	Sample analysis (i.e., virus RNA quantification and IgG ELISAs) was performed on blinded samples.

Reporting for specific materials, systems and methods

We require information from authors about some types of materials, experimental systems and methods used in many studies. Here, indicate whether each material, system or method listed is relevant to your study. If you are not sure if a list item applies to your research, read the appropriate section before selecting a response.

Materials & experimental systems

n/a	Involved in the study	
☐	☒	Antibodies
☒	☐	Eukaryotic cell lines
☒	☐	Palaeontology and archaeology
☐	☒	Animals and other organisms
☒	☐	Clinical data
☒	☐	Dual use research of concern
☒	☐	Plants

Methods

n/a	Involved in the study	
☒	☐	ChIP-seq
☒	☐	Flow cytometry
☒	☐	MRI-based neuroimaging

Antibodies

Antibodies used	An HRP-conjugated goat anti-guinea pig IgG(H+L) secondary antibody was used for ELISAs. The antibody was obtained from KPL (Cat. No. 14-17-06).
Validation	The above antibody was validated by the manufacturer for specificity and lot-to-lot consistency using an in-house ELISA assay.

Animals and other research organisms

Policy information about studies involving animals; ARRIVE guidelines recommended for reporting animal research, and Sex and Gender in Research

Laboratory animals	Female Hartley guinea pigs, 200-300 g, were used for this study.
Wild animals	This study did not involve wild animals.
Reporting on sex	Only female animals were used in this study.
Field-collected samples	This study did not involve samples collected from the field.
Ethics oversight	All animal experiment protocols in this study were reviewed and approved by the Animal Care Committee at the Canadian Science Centre for Human and Animal Health (CSCHAH), Winnipeg, Manitoba, Canada, in accordance with guidelines from the Canadian Council on Animal Care (CCAC).

Note that full information on the approval of the study protocol must also be provided in the manuscript.
